# Leakage pressures for gasketless superhydrophobic fluid interconnects for modular lab-on-a-chip systems

**DOI:** 10.1038/s41378-021-00287-6

**Published:** 2021-09-02

**Authors:** Christopher R. Brown, Xiaoxiao Zhao, Taehyun Park, Pin-Chuan Chen, Byoung Hee You, Daniel S. Park, Steven A. Soper, Alison Baird, Michael C. Murphy

**Affiliations:** 1 Center for Bio-Modular Multi-Scale Systems, Louisiana State University, Baton Rouge, LA 70803 USA; 2 Department of Mechanical & Industrial Engineering, Louisiana State University, Baton Rouge, LA 70803 USA; 3 Department of Mechanical and Industrial Engineering, University of Toronto, Toronto, ON M5S 3GB Canada; 4 School of Mechanical Engineering, Kyungnam University, Changwon, South Korea; 5 Department of Engineering Technology, Texas State University, San Marcos, TX 78666 USA; 6 Department of Mechanical Engineering, The University of Kansas, Lawrence, KS 66045 USA; 7 Department of Chemistry, The University of Kansas, Lawrence, KS 66045 USA; 8 SUNY Downstate Stroke Center, University Hospital of Brooklyn, Brooklyn, NY 11203 USA

**Keywords:** Microfluidics, Nanoparticles

## Abstract

Chip-to-chip and world-to-chip fluidic interconnections are paramount to enable the passage of liquids between component chips and to/from microfluidic systems. Unfortunately, most interconnect designs add additional physical constraints to chips with each additional interconnect leading to over-constrained microfluidic systems. The competing constraints provided by multiple interconnects induce strain in the chips, creating indeterminate dead volumes and misalignment between chips that comprise the microfluidic system. A novel, gasketless superhydrophobic fluidic interconnect (GSFI) that uses capillary forces to form a liquid bridge suspended between concentric through-holes and acting as a fluid passage was investigated. The GSFI decouples the alignment between component chips from the interconnect function and the attachment of the meniscus of the liquid bridge to the edges of the holes produces negligible dead volume. This passive seal was created by patterning parallel superhydrophobic surfaces (water contact angle ≥ 150°) around concentric microfluidic ports separated by a gap. The relative position of the two polymer chips was determined by passive kinematic constraints, three spherical ball bearings seated in v-grooves. A leakage pressure model derived from the Young–Laplace equation was used to estimate the leakage pressure at failure for the liquid bridge. Injection-molded, Cyclic Olefin Copolymer (COC) chip assemblies with assembly gaps from 3 to 240 µm were used to experimentally validate the model. The maximum leakage pressure measured for the GSFI was 21.4 kPa (3.1 psig), which corresponded to a measured mean assembly gap of 3 µm, and decreased to 0.5 kPa (0.073 psig) at a mean assembly gap of 240 µm. The effect of radial misalignment on the efficacy of the gasketless seals was tested and no significant effect was observed. This may be a function of how the liquid bridges are formed during the priming of the chip, but additional research is required to test that hypothesis.

## Introduction

Many different configurations of modular microfluidic systems have been reported. Example architectures include stack modular^1–6^, bus modular^7–12^, sectional modular^13–15^, and hybrid systems^3,16^. Modular architectures offer a variety of benefits including the ability to build complex systems using simple building blocks, the one-to-one mapping of functional elements and physical components, the use of standardized physical connections between component parts, and enabling low-cost mass production by using similar components in different applications. Standardized physical connections come in the form of optical, electrical, geometric, and fluidic interfaces. The most important of these standardized connections is the fluidic interconnect. It is a passage used to transport a liquid analyte, containing both mass and information, between component chips (chip-to-chip) and to/from the microfluidic system (world-to-chip). A range of materials^17–20^, orientations^21,22^, and length scales^23–26^, have been reported and various review articles have evaluated the field^27,28^. Most designs rely on some form of physical contact between the interconnect components to seal the connection. This physical contact comes from either strain induced in one or more elastic interconnect components or by eliminating the gap between interconnect components with a filling material with a different stiffness, such as polydimethylsiloxane (PDMS) or epoxy. As a consequence, each additional fluidic interconnect adds a kinematic constraint to the microfluidic system.

Two components have six relative degrees of freedom (DOF), translation along and rotation about each axis of a Cartesian coordinate system. Each assembly feature or interconnect removes one or more of those DOF. The use of multiple interconnects leads to either an over-constrained system, where additional force is required to assemble component chips, or an under-constrained system, when additional clearances are included around alignment features to ensure assembly^29^. In the over-constrained case, the additional assembly force is proportional to the number of interconnects and the strain it induces in the final assembly creates both indeterminate dead volumes and misalignment between the chip modules. For under-constrained assemblies, the clearances also lead to unpredictable dead volumes and misalignment between modules, because manufacturing tolerances lead to variation in the clearance geometry from chip-to-chip. The problem of over-constraint artificially limits the number of fluidic interconnects between modules. If the constraint is decoupled from the design of the interconnect, the development of highly multi-component modular microfluidic systems is enabled.

Many applications, including inkjet printing, droplet microfluidics, droplet chemical reactors^30^, and rheometry^31,32^, depend on either droplet–surface or droplet–droplet interactions. Underlying the behavior of these systems is a dependence on the formation and stability of liquid bridges, which are thin filaments of fluid that span the gaps between droplets or droplets and surfaces.

A gasketless, proximity-based fluidic interconnect that uses parallel superhydrophobic surfaces (contact angle ≥150°) to passively form a liquid bridge suspended between two concentric through-holes separated by a gap is characterized. The gasketless, superhydrophobic fluid interconnect (GSFI) decouples alignment between chip-level modules from the interconnect design. This enables multiple simultaneous connections, requires no additional components such as a gasket or an o-ring, and can be manufactured using established mass production techniques such as injection molding, spin coating, and polishing. To incorporate the GSFI into chip-based modules, passive chip-level kinematic alignment structures^33^ are used to align and set the gap between adjacent through-holes in the modular component chip assemblies.

Analytical models based on the capillary forces for the gasketless interconnect were used to estimate the leakage pressure for the GSFI as a function of the geometric and surface parameters. The leakage pressures of GSFIs on prototype devices were measured and compared to the leakage pressures estimated by models to assess the validity of the models and the feasibility of the approach.

## Results and discussion

### Foundational concepts

**Fig. 1:**
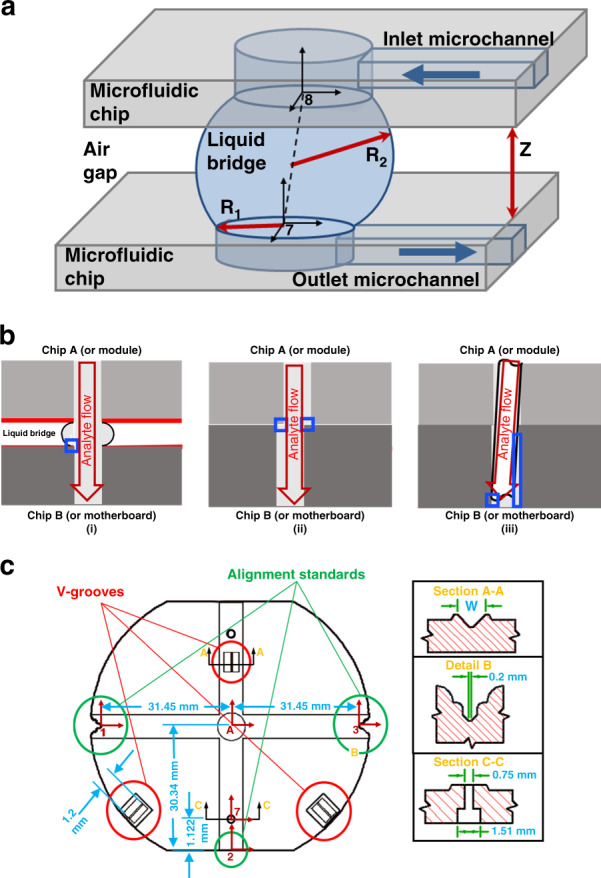
Schematic of Gasketless Superhydrophobic Fluidic Interconnect (GSFI) Potential Dead Volumes in Different Types of Fluid Interconnects and the Test Chip Geometry. **a** A schematic of the gasketless interconnect transporting fluid from one component chip to another. The schematic indicates the location of the leakage pressure model’s parameters of the gap, *Z*, the through-hole radius of curvature, *R*_1_, and the meniscus radius of curvature, *R*_2_. The schematic also shows that coordinate frames 7 and 8 are centered on the inlet and outlet of the gasketless seal. **b** Schematics of the effect of manufacturing variance on the dead volume between two fluidic chips or a module and a motherboard, for: (i) the GSFI, with the liquid bridge not attached at the edge of the through-hole and the associated dead volume defined by the blue square; (ii) a simple, misaligned through-hole, with the dead volume identified by the blue squares; (iii) a capillary tube used as a connector with manufacturing variation, or other inserts, inserted in a misaligned through-hole and the dead volume shown by the blue rectangles. **c** A schematic of the geometry of an injection-molded sample chip. V-groove/ball bearing kinematic alignment structures (Section A-A) were used to passively constrain the samples six degrees of freedom with six-point contacts. The alignment structures widths were dependent upon the designed gap between the sample chips (Table 1) and manufacturing variation. Alignment standards were used to measure chip misalignment (Detail B). Injection-molded through-holes with a backside counter bore (Section C-C) were used to interface with a press-fit world-to-chip connection which served as an inlet or outlet to the chip assembly. Coordinate frames for misalignment analysis are shown with the base frame for the chip (Frame A), the alignment standards (Frames 1, 2, and 3), and through-hole (Frame 7).

The GSFI (see schematic in Fig. 1a) uses a potential energy barrier induced by capillary forces as a seal. The potential energy barrier emanates from the minimization of the system’s interfacial and mechanical potential energy. Capillary forces include both surface tension forces and Young–Laplace forces^34,35^. Surface tension forces are proportional to the surface tension of the liquid–vapor interface and the length of the triple line created by the intersection of the liquid–vapor, solid–liquid, and solid–vapor interfaces. The Young–Laplace forces are proportional to the mean curvature of the liquid–vapor interface and the surface tension of the liquid–vapor interface.

The investigation of the GSFIs builds upon prior investigations into capillary forces, liquid bridge stability, and the stability of the pendant drop. The pioneers in the study of capillarity were Young^36^ and Laplace^37^. Understanding the nature of capillary forces led to the investigation of static liquid bridges and their stability. Static stability depends primarily on potential energy. There are two pedagogic classes of static liquid bridge stability problems: (1) Constant pressure^38^ (pressure control^39^); and (2) constant volume^38^ (volume control^39^). Plateau^40^ first investigated capillary surfaces and predicted the stability of weightless liquid bridges^41^. Howe^42^ studied the stability of constant volume, weightless axisymmetric capillary surfaces. A review by Michael^39^ explored some of the significant experimental and theoretical developments in the understanding of meniscus stability. Chen^43^ investigated the limits of stability of capillary bridges between parallel and non-parallel surfaces. Lowry^38^ mapped the manifold of all stable and unstable equilibria for weightless axisymmetric static liquid bridges parameterized by liquid bridge volume, liquid bridge length, and small Bond numbers. Lowry^44^ presented a method for predicting the onset and stability character of non-axisymmetric modes in liquid bridges and drops. Slobozhanin^45^ calculated the stability margin for a subset of weightless axisymmetric static liquid bridge equilibrium configurations using the height of the local potential energy barrier.

Using the liquid bridge in the GSFI has the benefit of improving the control of dead volumes in assembled parts. All manufacturing processes have inherent variation, which over large numbers of components has a Gaussian distribution. This will introduce unswept dead volumes, where target materials can be trapped, as shown schematically in Fig. 1b. For the GSFI (Fig. 1b, i), the fluid path is determined by the attachment point for the liquid bridge which is a function of the hydrophobicity of the polymer and the superhydrophobic film. The coating can be spin-coated up to the edge of the through-hole^46^. For a conventional assembly of a module and a motherboard or two fluidic chips with misalignment between a pair of through-holes the dead volume is much larger, matching the size of the misalignment (Fig. 1b, ii). Inserting a capillary tube or other form of insert into a misaligned through-hole, see Fig. 1b, iii, to facilitate the transfer of fluids will have manufacturing tolerance issues in the finish of the ends of the capillary or the angle of insertion that can result in large dead volumes also.

For the GSFI, Young–Laplace forces dominate the surface tension forces *(*Δ*P*_Laplace_ ≫ Δ*P*_Surface_) when connecting micro- and nanochannels^35^. The Young–Laplace forces are modeled by the Young–Laplace equation^47^ (Eq. 1):

1 $${\Delta} P = \gamma \left( {\frac{1}{{R_1}} + \frac{1}{{R_2}}} \right) = 2\gamma H$$

where Δ*P* is the difference in pressure across the liquid–vapor interface, *γ* is the surface tension of the liquid–vapor interface, *R*_1_ is the radius of the through-hole, *R*_2_ is the radius of curvature of the meniscus, which is orthogonal to *R*_1_, and H is the mean surface curvature.

A static leakage pressure model was derived from the Young–Laplace equation to predict the leakage pressure of the interconnect for different gaps^48^. The interconnect was assumed to leak under the following conditions: the liquid bridge reaches the static water contact angle of the superhydrophobic surface; the static water contact angles of the top and bottom surfaces are equal; the through-holes are identical, concentric cylinders; the opposing surfaces are perfectly parallel, and a semi-circular arc approximates the shape of the meniscus. Under these assumptions, the static leakage pressure of the gasketless seal is reduced to Eq. 2 from Eq. 1:

2 $${\Delta} P = \gamma \left( {\frac{1}{{R_1}} - \frac{{2\cos \theta _{\mathrm{c}}}}{Z}} \right)$$

where Δ*P* is the leakage gauge pressure of the interconnect, is the surface tension of the liquid–vapor interface, *R*_1_ is the radius of the through-holes, *θ*_c_ is the static water contact angle of the surface, and *Z* is the gap (Fig. 1a).

The sample chip geometry, shown schematically in Fig. 1c, consisted of three v-grooves for exact kinematic alignment, alignment standards for assessing the alignment accuracy of the assemblies^29^, elevated isolation zones to facilitate the creation of superhydrophobic surfaces around the fluid ports, and injection-molded through-holes with backside counter bores for fluidic transfer between chips. An assembly consisted of two sample chips with ball bearings seated in the v-grooves to define the gap. A summary of the range of v-groove and gap dimensions is presented in Table 1.

**Table 1:** The v-groove widths necessary to achieve different nominal gap distances for chip assemblies using a 0.794 mm diameter ball bearing.

Nominal gap distance (*z*)	Width at the base of the v-groove (*W*)
5 μm	1.126 mm
15 μm	1.116 mm
25 μm	1.106 mm
35 μm	1.096 mm
45 μm	1.086 mm
55 μm	1.076 mm
65 μm	1.066 mm
75 μm	1.056 mm
85 μm	1.046 mm
95 μm	1.036 mm
105 μm	1.026 mm
115 μm	1.016 mm
125 μm	1.006 mm
135 μm	0.996 mm

The isolation areas around the through-holes were elevated above the substrate to aid polishing prior to deposition of the superhydrophobic surfaces. These mitigated the effects of manufacturing defects, such as burrs, on the flatness of the kinematic alignment structures. The isolation areas were 20 μm above the sample surface and formed a cross that spanned the sample chips.

The three v-grooves served as part of the passive kinematic alignment structures that set the assembly gap distance and concentrically aligned the through-holes. The v-grooves had a 45° taper and their depths and widths were dependent on the desired gap. Three, grade 5, 0.794 mm (1/32 in) diameter silicon nitride (Si_3_N_4_) ball bearings (Boca Bearing Company, Boynton Beach, FL), were used. The three passive kinematic alignment structures constrained all six degrees of freedom of each component chip using a set of six-point contacts^29,49,50^. The kinematic alignment standards were incorporated to serve as reference structures for comparing the dimensions of the assemblies. The left and right alignment standards (Fig. 1c) were the same dimensions as used by You et al.^33,51^, and had a 200 μm wide flat as the measurement standard.

The other half of the planar misalignment was measured using a tool mark embedded on the sample’s main flat, which was centered on the through-hole.

Homogeneous coordinate transformation matrices were used to relate the chip and assembly geometry to the sample assembly’s measured offsets and to enable the calculation of the radial misalignment. The coordinate transformation matrices consisted of a 3 × 3 rotation matrix, *R*, which describes the orientation of a feature coordinate frame with respect to the base coordinates, and a 3 × 1 translation vector, *p*, which locates the origin of the feature coordinates in the base coordinate frame. Equation 3 shows the general form of the nominal coordinate transformation matrix.

3 $$T = \left[ {\begin{array}{*{20}{c}} R & p \\ {0^T} & 1 \end{array}} \right]$$

On each chip, homogeneous coordinate transformation matrices describe the position and orientation of the left, right, and top alignment standards to the base coordinate frame at the center of the chip. For a pair of assembled test chips, a coordinate transformation matrix defines the relative orientation and position of the chip base coordinate frames. Combining the chip and assembly transformations with the offset measurements resulted in the linear system shown in Eq. 4. A detailed description of the derivation is given in the Supplemental Information.

4 $$\left[ {\begin{array}{*{20}{c}} 1 & 0 & {\overline {A2} } \\ 0 & 1 & { - \overline {A3} } \\ 0 & 1 & {\overline {A3} } \end{array}} \right]\left[ {\begin{array}{*{20}{c}} {\varepsilon _x} \\ {\varepsilon _y} \\ {\delta \theta _z} \end{array}} \right] = \left[ {\begin{array}{*{20}{c}} {\overline {{{{\boldsymbol{x}}}}_{\mathbf{25}}} } \\ {\overline {{{{\boldsymbol{y}}}}_{\mathbf{14}}} } \\ {\overline {{{{\boldsymbol{y}}}}_{\mathbf{36}}} } \end{array}} \right]$$

where $\overline {A2}$ is the distance from a chip’s center to the chip’s top alignment standard (30.34 mm), $\overline {A3}$ is the distance from a chip’s center to the chip’s left or right alignment standard (31.45 mm), $\overline {{{{\boldsymbol{x}}}}_{\mathbf{25}}}$ is the sample mean of the offset measurements at the top alignment standard, $\overline {{{{\boldsymbol{y}}}}_{\mathbf{14}}}$ is the sample mean of the offset measurements at the left alignment standard, $\overline {{{{\boldsymbol{y}}}}_{\mathbf{36}}}$ is the sample mean of the offset measurements at the right alignment standard, *ε*_*x*_ is the *x*-component of the offset between both chips’ centers, *ε*_*y*_ is the *y*-component of the offset between both chips’ centers, and *δθ*_*Z*_ is the relative rotation between the top and bottom chips. A least-squares solution can be found for Eq. 4 and translated to the coordinate frames connecting both chips through-holes^29,52^.

The resulting $\widehat {\varepsilon _x}$ and $\widehat {\varepsilon _y}$ translation components give the *x*- and *y*-components of the radial misalignment between the through-holes. The radial misalignment is determined by Eq. 5:

5 $$\delta = \sqrt {\widehat {\varepsilon _x}^2 + \widehat {\varepsilon _y}^2}$$

where *δ* is the least-squares radial misalignment, $\widehat {\varepsilon _x}$ is the *x*-component of translation, and $\widehat {\varepsilon _y}$ is the *y*-component of translation.

### Design space

Dimensional analysis was used to determine the flow conditions where the static leakage pressure model is appropriate as a dynamic model. This flow regime is defined by conditions where surface tension forces dominate viscous, inertial, and gravitational forces. This regime was found by using a set of dimensionless parameters including the capillary number, the Weber number, and the Bond number. The capillary number (Ca) compares the viscous forces to the surface tension forces and is given by ${\mathrm{Ca}} = \frac{{\mu U}}{\gamma }$, where *μ* is the dynamic viscosity, *U* is the fluid velocity, and *γ* is the interfacial tension between the two fluids. The Weber number (We) is the ratio of inertial forces to surface tension forces and is given by ${\mathrm{We}} = \frac{{\rho U^2L}}{\gamma }$, where *ρ* is the density and *L* is the characteristic length of the flow. The Bond number (Bo) is the ratio of gravitational forces to surface tension forces and is given by ${\mathrm{Bo}} = \frac{{{\Delta} \rho gL^2}}{\gamma }$, where Δ*ρ* is the difference in density between the fluid and air, and *g* is the acceleration due to gravity.

**Fig. 2:**
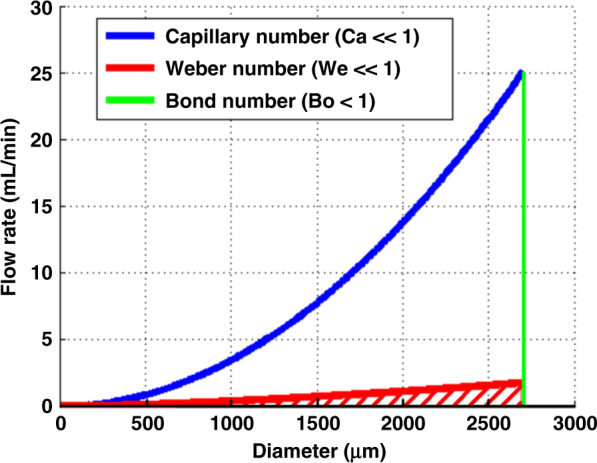
Dimensionless Design Space for GSFI’s. Under the conditions of We ≪ 1, Ca ≪ 1, and Bo  <  1, the static maximum pressure model is appropriate as a dynamic model. The acceptable range of through-hole diameters and flow rates that meet these criteria are shown in the red cross-hatched area.

The feasible design space for the GSFI’s are the diameters and flow rates that satisfy the conditions of Ca ≪ 1, We ≪ 1, and Bo ≪ 1, were much less than one was assumed to correspond to the dimensionless numbers being <0.001. These are the interior of the red, cross-hatched triangular region in Fig. 2. Under these conditions, the static leakage pressure model approximates the dynamic behavior of the fluid. The leakage test experiments were performed at combinations of acceptable flow rates and diameters chosen from Fig. 2. Using the criteria from Fig. 2, the maximum acceptable flow rate for the leakage test experiment’s 750 μm diameter through-holes was 260 μL/min. This maximum acceptable flow rate was much greater than the maximum flow rate experienced during the experiment’s priming procedure (~0.3 μL/min). To put the design space in the context of a microfluidic device, Chen et al. described a 20 cycle continuous-flow polymerase chain reaction (CFPCR) microfluidic chip^53^ that utilized a microchannel with a cross-section of 20 μm by 40 μm and a length of 193 mm that transitioned to a cross-section of 40 μm by 40 μm for a length of 193 mm. If the input and/or output of this microfluidic chip utilized a GSFI, the maximum acceptable flow rate would be ~1 μL/min. The CFPCRsempirically determined optimal flow rate of 48 nL/min^53^ fits within the feasible design space of the GSFI’s.

**Fig. 3:**
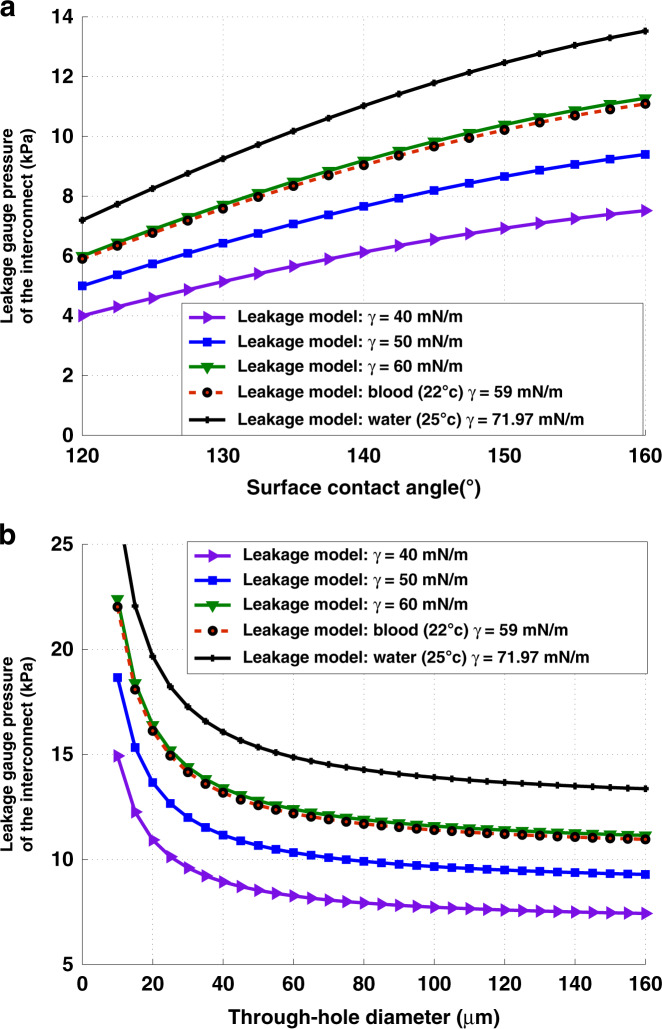
The Young–Laplace model of the estimated leakage pressures for the liquid bridge. **a** Variation of the leakage pressure as a function of the surface contact angle and surface tension of the fluid passing through the interconnect. **b** Change in the leakage pressure as a function of the through-hole diameter and fluid surface tension. The leakage pressure is essentially constant for larger diameters.

The parameter space of the gasketless seal was evaluated to show its efficacy with different levels of hydrophobicity, liquid analytes, and through-hole diameters. Figure 3a demonstrates the effect of water contact angle and surface tension of the liquid analyte on the leakage pressure of the GSFI’s for a gap of 10 μm and a through-hole diameter much larger than the gap. It shows that the decrease in leakage pressure for fluids close to the surface tension of deionized water, such as whole blood, can be effectively compensated for by increasing the water contact angle. Figure 3b evaluates the effect of the through-hole diameter and surface tension of the liquid analyte on the leakage pressure of the GSFI assuming a gap of 10 μm and a water contact angle of 150°. Large variations in the surface tension from the surface tension of water can be compensated for by decreasing the through-hole diameter.

### Fabrication results

**Fig. 4:**
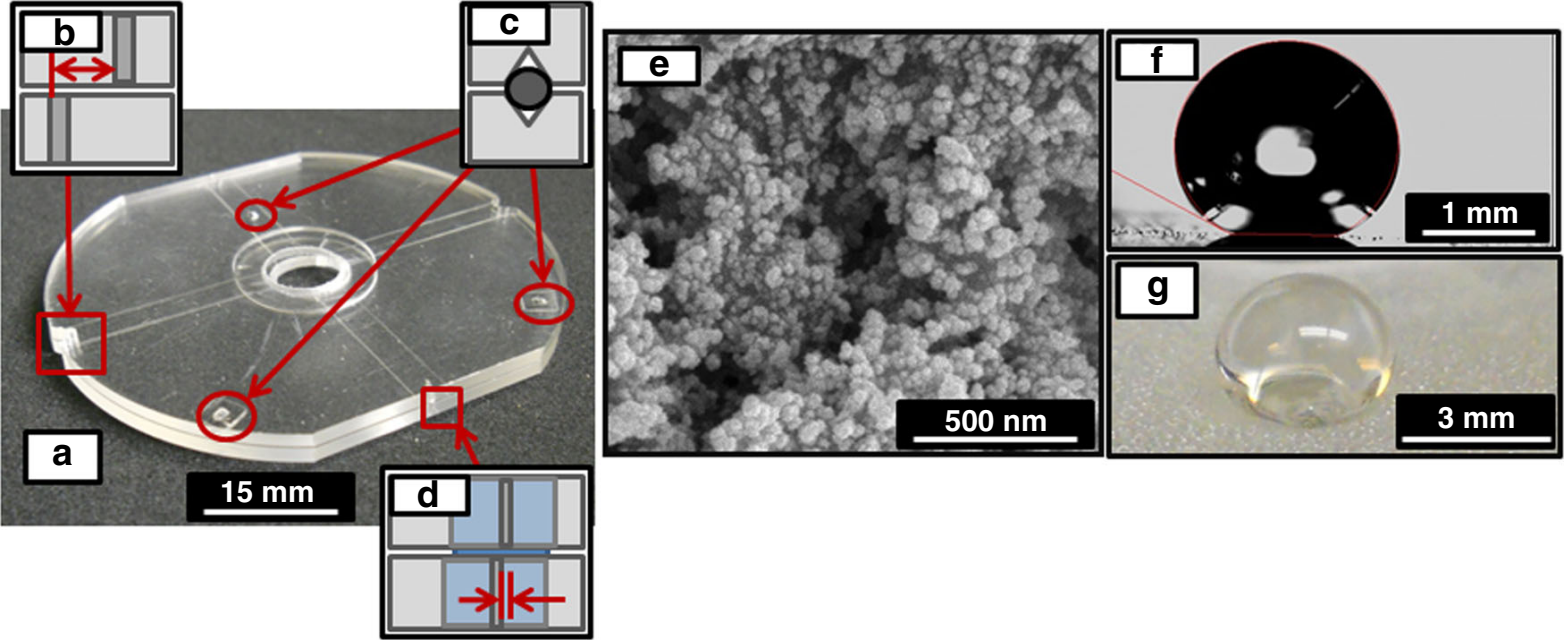
Sample Test Chip and Superhydrophobic Coating. **a** An assembled chip assembly without the superhydrophobic surface for visual clarity. The interconnect assembly features **b** a pair of alignment standards, **c** three v-groove/ball bearing passive kinematic alignment structures, and **d** concentric 750 μm diameter injection-molded through-hole with tool mark to measure alignment. **e** Top view of Hydrobead-P on a COC sample chip imaged using a scanning electron microscope (SEM) (FEI Quanta3d FEG, Helios Nanolab, Hillsboro, OR), sputter-coated with 80 nm of platinum. **f** Static contact angle measurement of 152° using the sessile drop technique with a 5–6 μL droplet on a VCA Optima (Billerica, MA). **g** Picture of the water droplet on Hydrobead-P-coated COC sample chip.

The leakage pressure and corresponding assembly gap were measured for 99 assemblies from four different mold inserts (Mold A, Mold B, Mold C, and Mold D) using two different loading conditions, clamped and unclamped. A representative test chip assembly is shown in Fig. 4a without the superhydrophobic coating to enable visualization of the critical features, which are highlighted in the inserts Fig. 4b–d. Figure 4b shows the alignment standards used to quantify lateral misalignment. The passive alignment structures including the v-grooves on each chip and the ball bearings inserted in the grooves are presented in Fig. 4c. The nominally concentric through-holes are highlighted in Fig. 4d. The ball bearings were selected for the alignment structures to enable a broad range of assembly gaps with a small number of mold inserts. In practice, both the v-grooves and alignment structures will be injection-molded^29^ or hot-embossed in the polymer directly^46,50,54^.

The results of the superhydrophobic coating are given in Fig. 4e–g. During spin-coating, Hydrobead-P filled one or both of the tool mark alignment standards on the majority of the assemblies, making 53 unavailable for misalignment analysis, but radial misalignment was calculated for 46 sample assemblies. Figure 4d shows a scanning electron microscope (SEM) (FEI Quanta3d FEG, Helios Nanolab, Hillsboro, OR) image of the Hydrobead-P® surface, demonstrating the typical multiscale roughness of the films. The result of this is illustrated in Fig. 4e, which is a water contact angle measurement using the sessile drop method^47^ (VCA Optima, Billerica, MA) of Hydrobead-P® on a sample chip’s surface. A water droplet on a sample chip coated with Hydrobead-P® is presented in Fig. 4f.

### Leakage pressure measurements

**Fig. 5:**
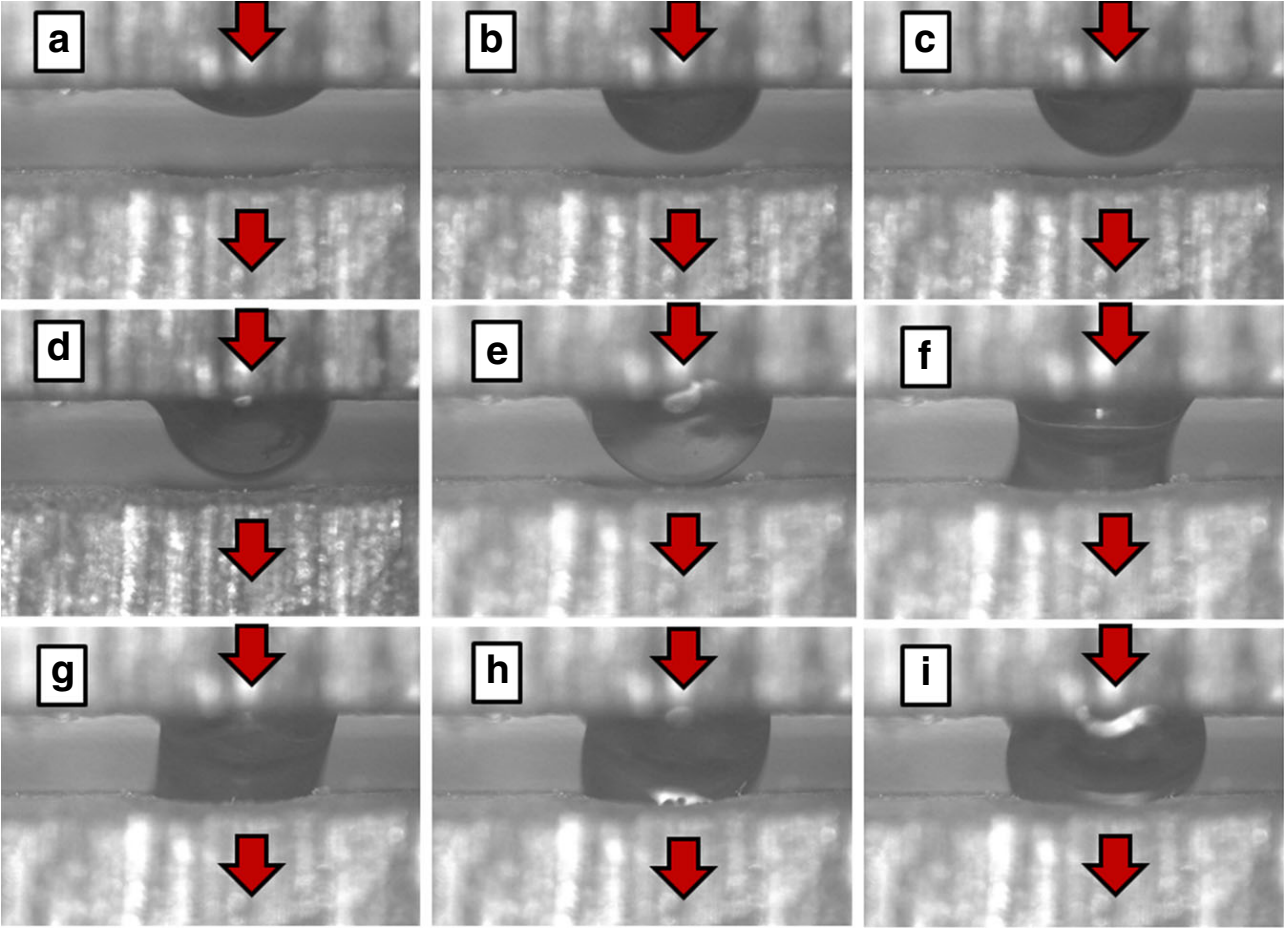
Experimental Formation of a Liquid Bridge to Establsh an GSFI. **a**–**i** Side view of the formation of the liquid bridge across a gasketless interconnect from proof-of-concept experiments^48^ using a Nikon Measurescope MM-11 (Melville, NY) and a ×10 objective. The images show deionized water, dyed with red food coloring, growing from the top chip inlet hole as an axisymmetric pendant drop (**a**–**e**), transitioning to a liquid bridge (**f**), and transporting liquid across the gasketless seal (**g**–**i**). The hole diameters were 800 μm and the gap between the chips was 600 μm.

The formation, or priming, of the GSFI during the proof-of-concept experiments^48^ is represented by the sequence of images in Fig. 5a–i. The priming process had two stages: the growth of an axisymmetric non-wetting pendant drop suspended from a circular orifice and the transition from the pendant drop to a liquid bridge. The dynamics and stability of axisymmetric pendant drops have been studied^39,55,56^. However, the physics of the gasketless seal’s transition from a growing non-wetting axisymmetric pendant drop to a liquid bridge requires further investigation. It was observed that the transition was associated with the liquid changing from a positive gauge pressure, with a convex curvature of the liquid bridge (Fig. 5e), to a negative gauge pressure, corresponding to a concave curvature of the liquid bridge (Fig. 5f). The proof-of-concept experiments^48^ demonstrated that both stages of the priming process were stable for flow rates from 0 to 0.3 mL/min and pressures from 0 to 0.3 kPa.

**Fig. 6:**
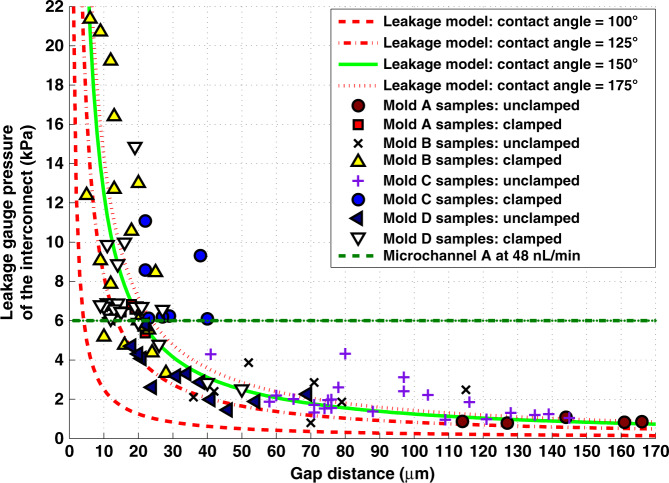
Measured Leakage Pressures as a Function of the Gap Distance Compard to Estimated Young-Laplace Pressures. A plot of the data from 99 sample assemblies representing the relationship between maximum pressure of the interconnect assembly and the gap compared to the maximum pressure model using a contact angle of 150°, and the pressure drop across Microchannel A at a flow rate of 48 nL/min. The maximum measured gap was used.

The leakage pressure experiment was an example of a pressure-controlled, static, short-liquid bridge stability problem. The leakage pressures of the prototype GSFIs were quantified for a range of assembly gaps and compared to the predictions from the leakage model (Eq. 2). The maximum measured leakage pressure for a GSFI was 21.4 kPa (3.1 psig), which corresponded to a measured mean assembly gap of 3 μm. The mean assembly gap was calculated from ten side view measurements of the gap distance using a Nikon Measurescope MM-11 (Melville, NY) with a Diagnostic Instruments, Inc microscope camera (Sterling Heights, MI) with SPOT advanced imaging software, and a QUADRA-CHEK 2000 (Metronics, Schaumburg, IL) with a ×50 objective. The lowest measured leakage pressure was 0.5 kPa (0.073 psig) for a device with a mean assembly gap of 240 μm. In Fig. 6, the leakage pressure is shown as a function of the measured assembly gap for 99 experimental assemblies and compared to the leakage pressure model. Measured rupture pressures were within ±50% of the leakage pressures estimated using Eq. 2 assuming a water contact angle of 150° 78% of the time. This gives confidence that the model is appropriate for designing GSFI’s. To give a sense of whether the GSFI can withstand normal operating pressures in microfluidic devices, the pressure drop across a microchannel with a cross-section of 20 μm by 40 μm for a length of 193 mm that transitions to a cross-section of 40 μm by 40 μm for a length of 193 mm at a flow rate of 48 nL/min are shown in Fig. 6. Microchannel A’s pressure drop is similar to the pressure drop from Chen et al. 20 cycle continuous-flow polymerase chain reaction (CFPCR) microfluidic chip^53^ at the optimal flow rate. GSFI’s with gaps <20 μm would not leak under the microfluidic driving pressures required for Microchannel A.

A wide range of chip-to-chip interconnect technologies were demonstrated with maximum pressures ranging from 6.9^26^ to 608 kPa^3^, including gaskets, o-rings, or wax^2,3,12,18,20,21,57,58^; interference fits^12,59,60^; eutectic bonds; and compression of double-sided tape^4^. The reported pressures, and consequently flow rates, are higher than the measured maximum pressure 21.4 kPa, but there are two critical differences. None of the methods are truly reversible and, if the chips contain more than one interconnect, the chips will be over-constrained and have unmeasurable dead volume, unlike the GSFI’s which are inherently reversible and exactly constrained with fully predictable dead volumes.

**Fig. 7:**
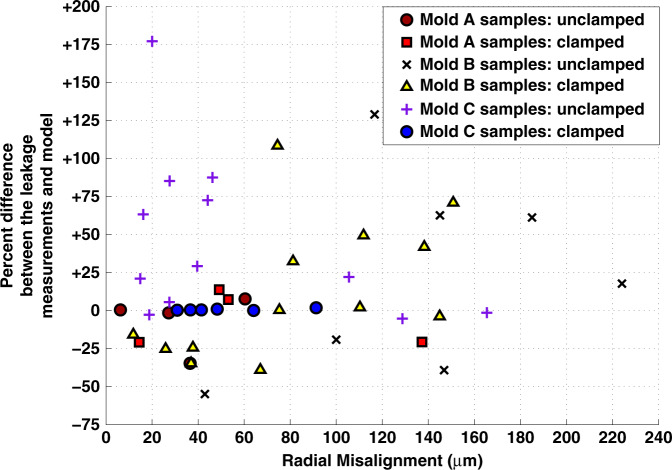
Experimental Sensitivity of Leakage Pressures to Radial Misalignment. A scatter plot of the radial misalignment of 46 sample assemblies against the percent difference between the measured leakage pressure and the model.

The radial misalignment was measured for 46 of the assemblies to elucidate its effect on the deviation of the rupture pressure measurements from the values estimated by the model. A scatter plot of the percent difference between the measured pressure and the model against the radial misalignment is shown in Fig. 7. No relationship was observed between the percent difference between the rupture measurements and the model and the radial misalignment. The tolerance for misalignment is hypothesized to be a function of the formation of the liquid bridge. During the priming of the system to form the liquid bridge, a droplet expands from the inlet side of the GSFI until it contacts the edge of the through-hole at the exit and is pinned to the edge at that point^30^. Because the gaps and the misalignments in the assemblies tested were small relative to the diameter of the through-holes, stable liquid bridges were formed in all cases. The misalignment data are detailed in the Supplementary Information.

Since the liquid bridge is pinned at the edges of the through-holes by the superhydrophobic surface, there is a minimal dead volume in the gasketless seal. This is driven by the proximity of the superhydrophobic surface to the edge, which is a function of the method of creating the superhydrophobic surface and should be considered in the development of any alternative fabrication processes. Similar results for liquid bridge attachment at the perimeter of a through-hole have been reported with a different transparent superhydrophobic coating by Zhao et al. who hypothesized that the liquid bridge attachment to the perimeter of the through-hole was also affected by the hydrophilic properties of the polymer in the through-hole^46,61^.

**Fig. 8:**
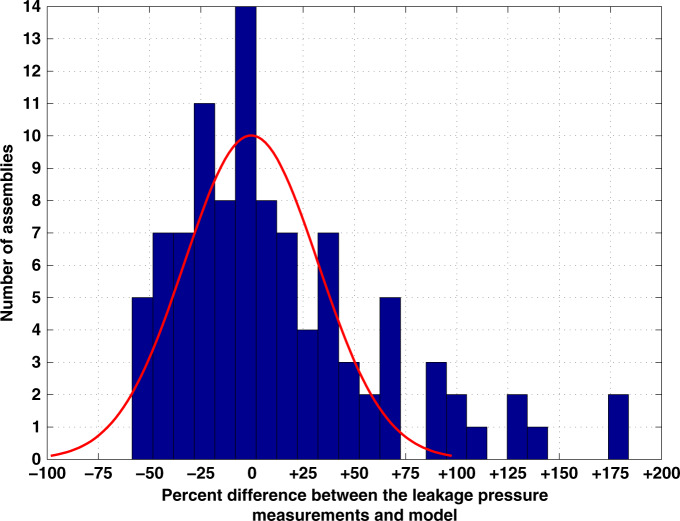
Distribution of Leakage Pressure Variation. A histogram of the percent difference between the measured leakage pressure measurements and the pressure predicted by the leakage pressure model using the maximum measured gap and a contact angle of 150°. A normal distribution based on the data for the ±75% range was transposed on top of the histogram. The data in the ±75% range has a mean of −0.3% and a standard deviation of 32.6%.

Figure 8 shows a histogram of the percent difference between the maximum leakage pressure measurements and the leakage pressure estimated by the model. From −75% to +75% differences, the distribution appears to be Gaussian with a mean of −0.3% and standard deviation of 32.6%, as would be expected from the Central Limit Theorem. The divergence from a Gaussian distribution for higher pressure differences may represent cases where the actual gap was smaller than observed during the measurement. The side view measurements of the gap that utilized an optical microscope with digital readout relied on the assumption that the assembly gap at the edge was the same as the gap at the through-hole. This assumption was necessitated by the optical microscope’s depth of focus, which was less than the 1.122 mm distance at the center of the GSFI. This assumption was justified by measurements of the flatness of uncoated polished samples using an optical profilometer. Potential causes of smaller gaps include (1) clamping forces causing curvature of the chips and increased the gap at the edge; (2) the interlocking of the superhydrophobic surface roughness around the through-hole creating a smaller apparent gap; and (3) the superhydrophobic surface preferentially building up around the through-hole.

## Conclusions

A broad spectrum of interconnecting designs has been documented in the literature. However, most of these add additional kinematic constraints that artificially limit the number of fluidic interconnects between component modules and generate unpredictable dead volumes either due to stresses arising from manufacturing variation in over-constrained systems or due to under-constraint. A novel microfluidic interconnect, the gasketless superhydrophobic fluid interconnect (GSFI), was proposed and the leakage pressure capability was evaluated. The GSFI uses capillary forces to seal the connection between two nominally, concentrically aligned through-holes in superhydrophobic surfaces separated by a gap. Polymer, passive kinematic alignment structures ensure the gap height, assembly with exact constraint, and the separation of the alignment and interconnect functions. A static model based on the Young–Laplace equation was developed to estimate the GSFI’s leakage pressure. Dimensional analysis was used to show the combination of velocities and through-hole diameters under which the static equation was appropriate as a dynamic model. A parametric analysis was used to demonstrate the efficacy of the GSFI with different hydrophobic surfaces, liquid analytes, and through-hole diameters. Leakage pressures were measured for 99, injection-molded COC sample gasketless assemblies using a commercially available superhydrophobic coating. The GSFI withstood maximum pressures up to 21 kPa and the measured leakage pressures were within ±50% of the rupture model for 78% of the maximum measured assembly gaps. This new interconnect technology enables the realization of highly parallel, modular systems with negligible dead volumes and repeatable, well-behaved assemblies. Similar results were obtained with different types of superhydrophobic surfaces, so the results should be generally applicable to polymer-based fluidic systems.

## Materials and methods

### Experimental methods

Two types of experiments were carried out to characterize the performance of the GSFIs: (1) Observation of the initiation and stability of the liquid bridge; and (2) Measurement of the leakage pressure for a nominal range of assembly gaps and comparison of the measured pressures with those estimated using the static leakage pressure model (Eq. 2).

Proof-of-concept experiments^48^ using deionized water mixed with red food coloring were used to observe the formation of the gasketless seal’s liquid bridge spanning a 600 μm gap set by a precision shim using an optical microscope with a ×10 objective.

### Sample chips

Sample chips for the measurement of the leakage pressures of the prototype GSFI’s were designed and manufactured to be assembled in pairs to create fluidic interconnects that could be easily interfaced with the instrumentation needed to measure the rupture pressure and the assembled gap.

The through-holes allowed the transport of the fluid to/from interconnected chips using the gasketless interconnect. Each through-hole had a diameter of 750 μm at the gasketless seal with its center located on the line of symmetry line of the sample chip and 1.122 mm from the edge of a flat to facilitate observation through a microscope. By injection molding the chips, repeatable placement and dimensions of the through-holes were ensured.

### Sample chip fabrication

All chips were injection-molded (Battenfeld BA 500/200 CDK-SE, Kottingbrunn, Germany) using four, single-cavity, prototype, injection mold dies. Chips were made from COC (Topas® 5013S-04, TOPAS Advanced Polymers, Florence, KY). All mold inserts were micromilled (MMP 2252, KERN Micro- and Feinwerktechnik GmbH & Co KG, Eschenlohe, Germany) in 353 brass (8948K21, McMaster-Carr, Atlanta, GA).

The four mold inserts were designed with different nominal v-groove dimensions for passive kinematic alignment to generate four different mean assembly gaps. The nominal gaps for each mold insert were: Mold A: 5 μm, Mold B: 25 μm, Mold C: 30 μm, Mold D: 50 μm. Through-holes were created in the chips using micromilled conical pillars that protruded from the mold cavity base and spanned the thickness of the parts. The base of the conical pillars served as the inlet or outlet of the GSFI in the molded part. The conical pillars had a diameter of 750 μm at the mold die base and a 14° taper along the pillars’ 1.87 mm height. The 14° taper was used to ensure that the injection-molded parts could be ejected from the mold die and to prolong the life of the mold insert possessing through-hole pillars.

After injection molding the chips, the reverse side of the through-holes, which was opposite the superhydrophobic surface, was counter bored to a depth of ~1.00 mm with a #53 jobber bit using a variable speed miniature drill press (MicroLux Tools, Berkeley Heights, NJ) because it was found empirically to provide the best press-fit connection with the 85.72 mm (3-3/8 in) length, 1.5875 mm (0.0625 in) OD, 0.76 mm (0.03 in ID) fluorinated ethylene propylene (FEP) tubing (1520, Upchurch Scientific, Oak Harbor, WA) that served as inlet and outlet world-to-chip connections to the sample assemblies. After drilling the counter bore, the samples were washed using detergent (Dawn Manual Pot and Pan Detergent, Procter & Gamble Professional, Cincinnati, OH) diluted in a bath of deionized (DI) water, and rinsed with DI water. The samples were then loaded into a convection oven (Model 1602, VWR, Radnor, PA) preheated to 70 °C, and dried for 4 h to prepare the samples for polishing.

Before applying the superhydrophobic coating to the samples, both sides of the COC chips were hand polished on a surface stone (DoAll, Des Plaines, IL) that had a flatness of 2.54 μm (0.0001 in). The front of the chips was polished with successively finer grit disks (600 grit, 9 μm grit, 3 μm grit) to mitigate the effects of manufacturing defects on the flatness of the kinematic alignment structures, through-holes, and alignment standards. The back-sides were polished to ensure a good vacuum seal between the chip and the vacuum chuck on the spin coating machine with a sequence of 600 grit and 9 μm grit materials. To clean the samples and prepare them for spin coating, they were washed using detergent diluted in deionized (DI) water, rinsed with DI water, and dried for 4 h in a convection oven set at 70 °C.

### Superhydrophobic coating: application and testing

A commercially available superhydrophobic coating, Hydrobead-P, was spin-coated onto the surface to achieve thin and uniform superhydrophobic surfaces. Hydrobead-P® (Hydrobead-P® “old formula”, Hydrobead, San Diego, CA) was selected as the superhydrophobic coating because of its low thickness (~2–16 μm, Supplementary Fig. S1), high contact angle uniformity, and its mean contact angle (~150°, Supplementary Fig. S2). Spin coating was selected as the application method because of its use as a mass production technique in microfabrication.

Hydrobead-P® was mixed as four parts of part-B (100 mL) to 1 part of part-A (25 mL) for each batch of chips. The solution was spin-coated onto the chips (Model SP100, Bidtec) at 1500 rpm for 30 s with ramp rates of 25 rpm/s. The samples were cured in a VWR 1602 (Radnor, PA) convection oven preheated to 100 °C for 1 h. The superhydrophobicity of the samples was assessed by dispensing deionized water droplets from a disposable pipette. The Hydrobead-P® application process was repeated up to two more times until all of the remaining samples were superhydrophobic.

### Assembly of the injection-molded chips

The interconnect prototypes were manually assembled from pairs of the Hydrobead-P®-coated chips. The variation introduced during manufacturing resulted in a distribution of the assembly gaps that enabled the stochastic testing of a larger range of the design space. The largest contributors to the manufacturing variation were the manual polishing, which removed material, and the superhydrophobic spin coating, which added material. To further extend the range of gaps tested, a clamped loading condition was introduced for some of the assemblies to achieve mean assembly gaps of <25.00 μm. Both clamped and unclamped loading conditions were used to assemble the chips. The unclamped assemblies used three medium binder clips (Staples, Framingham, MA) placed over the assembly’s kinematic alignment structures to hold the assembly while the Pacer Z-Poxy 5 min epoxy (Rancho Cucamonga, CA) was cured at four different locations around the edges of the assemblies. The clamped assemblies had an additional load applied across the backside through-holes using deep throat u-clamps (Harbor Freight Tools USA, Inc., Calabasas, CA) while the epoxy cured.

The interconnect assemblies were mated with inlet and outlet tubing using press-fit, world-to-chip, interconnects between the backside counter bores of the through-holes and the ends of two segments of 85.72 mm (3–3/8 in) length, 1.58 mm (0.0625 in) OD, 0.76 mm (0.03 in ID) FEP tubing (1520, Upchurch Scientific, Oak Harbor, WA). Pacer Z-Poxy (Rancho Cucamonga, CA) was used to reinforce the two world-to-chip connections. The other end of the assembly’s inlet FEP tube was assembled with a VacuTight^TM^ ferrule (P-840, Upchurch Scientific, Oak Harbor, WA) to connect to an upstream P-727 TEE (Upchurch Scientific, Oak Harbor, WA) junction on the leakage pressure experimental apparatus. The other end of the assembly’s FEP output tube was assembled with a super flangeless nut with ¼ −28 thread (LT-115, Upchurch Scientific, Oak Harbor, WA) and a super flangeless ferrule (P-250, Upchurch Scientific, Oak Harbor, WA) to connect to the downstream P-732 microfluidic ball valve (Upchurch Scientific, Oak Harbor, WA) on the leakage pressure experimental apparatus.

### Instrumentation

The pressure was automatically and incrementally increased across each GSFI assembly, while the interconnect pressure history was recorded until leakage occurred. A custom LabVIEW 2012 (National Instruments, Austin, TX) program running on a laptop computer was used to control and record the pressures. A schematic of the pressure control and measurement system and a more detailed description of the components are shown in the Supplementary Information.

### Experimental procedures

The leakage pressure experiments had two steps: (1) Measuring the leakage pressure of the interconnect assemblies; and (2) measuring the assembly gap and misalignment. The leakage pressure measurement had a start-up procedure, a priming procedure, and a leakage pressure testing procedure. The start-up procedure consisted of pressurizing the deionized water column, powering up the system, connecting the correct pressure sensor to the system, and opening LabVIEW and NI MAX (National Instruments, Austin, TX). Priming the system enabled the formation of the liquid bridge across the gap. More detailed descriptions can be found in the Supplementary Information.

A sampling rate of 5 kHz was set through the LabVIEW program for the system inputs. The ramp rate of the pressure controller set point was increased in steps of 0.014 kPa (0.002 psi) every 120 ms during the experiments. The driving pressure from the pressure controller was cut-off either automatically when the program sensed a drop in the running average of the pressure transducer signal or manually using Measurement and Automation Explorer (MAX) (National Instruments, Austin, TX) when leakage was observed visually. The running average used in the automatic cut-off was calculated over 100 ms segments. If the running average dropped more than the resolution of the pressure sensor between two successive segments, the program would shut down the experiment indicating leakage.

Gaps and radial misalignment between component chips in assemblies were measured and calculated to determine their effect on leakage pressure. A Nikon Measurescope MM-11 (Melville, NY) with Nikon ×10 and ×50 objectives was used in conjunction with a microscope camera (Diagnostic Instruments, Inc., Sterling Heights, MI) and a digital readout (Quadra-Chek 2000, Metronics, Schaumburg, IL) to measure the gap and radial misalignment. These measurements were gathered after the leakage pressure of the assemblies was determined. For each assembly, the gap was measured ten times within a range of ±400 μm from the gasketless seal’s center and the misalignment of the left and right alignment standards (Fig. 1c) were also each measured ten times. For a subset of the sample assemblies, the other half of the radial misalignment was measured ten times using the embedded tool mark on the flat. For that subset, the radial misalignment for each sample was calculated using a mathematical model that linked left, right, and top alignment standard data to the assembly geometry (Eq. 5). A detailed description of the method is located in the Supplementary Information.

## Supplementary information

Supplemental Material

## References

[1] Fettinger JC (1993). Stacked modules for micro flow systems in chemical analysis: concept and studies using an enlarged model. Sens. Actuators B Chem..

[2] Schoot BH (1993). Modular setup for a miniaturized chemical analysis system. Sens. Actuators B Chem..

[3] Miserendino S, Tai Y-C (2008). Modular microfluidic interconnects using photodefinable silicone microgaskets and MEMS O-rings. Sens. Actuators A Phys..

[4] Verpoorte EMJ (1994). Three-dimensional micro flow manifolds for miniaturized chemical analysis systems. J. Micromech. Microeng..

[5] Blazej RG, Kumaresan P, Mathies RA (2006). Microfabricated bioprocessor for integrated nanoliter-scale Sanger DNA sequencing. Proc. Natl Acad. Sci. USA.

[6] Kikutani Y (2002). Glass microchip with three-dimensional microchannel network for 2 x 2 parallel synthesis. Lab Chip.

[7] Shaikh KA (2005). A modular microfluidic architecture for integrated biochemical analysis. Proc. Natl Acad. Sci. USA.

[8] Chen YW (2012). Modular microfluidic system fabricated in thermoplastics for the strain-specific detection of bacterial pathogens. Lab Chip.

[9] Grodzinski P (2003). A modular microfluidic system for cell pre-concentration and genetic sample preparation. Biomed. Microdevices.

[10] Schabmueller CGJ (1999). Design and fabrication of a microfluidic circuitboard. J. Micromech. Microeng..

[11] Hashimoto M (2005). Ligase detection reaction/hybridization assays using three-dimensional microfluidic networks for the detection of low-abundant DNA point mutations. Anal. Chem..

[12] Gray BL, Collins SD, Smith RL (2004). Interlocking mechanical and fluidic interconnections for microfluidic circuit boards. Sens. Actuators A Phys..

[13] Yuen PK (2008). SmartBuild-a truly plug-n-play modular microfluidic system. Lab Chip.

[14] Rhee M, Burns MA (2008). Microfluidic assembly blocks. Lab Chip.

[15] Pepper, M. et al. Interconnecting fluidic packages and interfaces for micromachined sensors. *Sens. Actuators A Phys.* **134**, 278–285 (2007).

[16] Krulevitch P (2002). Polymer-based packaging platform for hybrid microfluidic systems. Biomed. Microdevices.

[17] Perozziello G, Bundgaard F, Geschke O (2008). Fluidic interconnections for microfluidic systems: A new integrated fluidic interconnection allowing plug’n’play functionality. Sens. Actuators B Chem..

[18] Gray BL (1999). Novel interconnection technologies for integrated microfluidic systems. Sens. Actuators A Phys..

[19] Igata E (2002). Interconnected reversible lab-on-a-chip technology. Lab Chip.

[20] Nittis V (2001). A high-pressure interconnect for chemical microsystem applications. Lab Chip.

[21] González C, Collins SD, Smith RL (1998). Fluidic interconnects for modular assembly of chemical microsystems. Sens. Actuators B Chem..

[22] Sabourin D (2010). One-step fabrication of microfluidic chips with in-plane, adhesive-free interconnections. J. Micromech. Microeng..

[23] Tsai JH, Lin L (2001). Micro-to-macro fluidic interconnectors with an integrated polymer sealant. J. Micromech. Microeng..

[24] Westwood SM, Jaffer S, Gray BL (2008). Enclosed SU-8 and PDMS microchannels with integrated interconnects for chip-to-chip and world-to-chip connections. J. Micromech. Microeng..

[25] Jaffer S, Gray BL (2008). Polymer mechanically interlocking structures as interconnects for microfluidic systems. J. Micromech. Microeng..

[26] Benett, W. & Krulevitch, P. A flexible package and interconnects for microfludic systems. In *SPIE BiOS ‘99* 8 (SPIE, San Jose, CA, 1999).

[27] Fredrickson CK, Fan ZH (2004). Macro-to-micro interfaces for microfluidic devices. Lab Chip.

[28] Temiz Y (2015). Lab-on-a-chip devices: How to close and plug the lab?. Microelectron. Eng..

[29] You B-H (2015). Assembly of polymer microfluidic components and modules: validating models of passive alignment accuracy. JMEMS.

[30] Curran K (2005). Liquid bridge instability applied to microfluidics. Microfluidics Nanofluidics.

[31] McKinley GH, Sridhar T (2002). Filament-stretching rheometry of complex fluids. Annu. Rev. Fluid Mech..

[32] Deganello D (2011). Level-set method for the modelling of liquid bridge formation and break-up. Comput. Fluids.

[33] You BH (2009). Passive micro-assembly of modular, hot embossed, polymer microfluidic devices using exact constraint design. J. Micromech. Microeng..

[34] Lambert, P. in *Microtechnology and MEMS* (eds Fujita, H. & and Liepmann, D.) (Springer Science+Business Media, LLC, New York, NY, 2010).

[35] Lambert P, Delchambre A (2005). Parameters ruling capillary forces at the submillimetric scale. Langmuir.

[36] Young T (1805). An essay on the cohesion of fluids. Philos. Trans. R. Soc. Lond..

[37] Laplace, P. S. *Traité de Mécanique Céleste* (Coureier, Paris, 1805).

[38] Lowry BJ, Steen PH (1995). Capillary surfaces: stability from families of equilibria with application to the liquid bridge. Proc. R. Soc. A Math. Phys. Eng. Sci..

[39] Michael DH (1981). Meniscus stability. Annu. Rev. Fluid Mech..

[40] Plateau, J. A. F. *Experimental and Theoretical Researches on the Figures of Equilibrium of a Liquid Mass Withdrawn from the Action of Gravity, Etc.* (Smithsonian Institution, 1863).

[41] Plateau, J. A. F. *Experimental and Theoretical Statics of Liquids Subject to Molecular Forces Only* (Gauthier-Villars, Paris, 1873).

[42] Howe, W. *Die Rotations-Flächen welche bei vorgeschriebener Flächengrösse ein möglichst grosses oder kleines Volumen enthalten* (Friedrich-Wilhelms-Universität zu Berlin, 1887).

[43] Chen T-Y, Tsamopoulos JA, Good RJ (1992). Capillary bridges between parallel and non-parallel surfaces and their stability. J. Colloid Interface Sci..

[44] Lowry BJ (2000). Modes of nonaxisymmetry in the stability of fixed contact line liquid bridges and drops. J. Colloid Interface Sci..

[45] Slobozhanin LA, Alexander JID, Patel VD (2002). The stability margin for stable weightless liquid bridges. Phys. Fluids.

[46] Zhao X (2020). Microfluidic gasketless interconnects sealed by superhydrophobic surfaces. JMEMS.

[47] Adamson, A. W. & Gast, A. P. *Physical Chemistry of Surfaces* (John Wiley & Sons, Inc, Hoboken, NJ, 1997).

[48] Brown, C. R. et al. Novel, gasketless, interconnect using parallel superhydrophobic surfaces for modular microfluidic systems. In *2011 ASME International Mechanical Engineering Congress and Exposition, Denver, CO* 5 (Denver, CO, 2011).

[49] Slocum A (2010). Kinematic couplings: a review of design principles and applications. Int. J. Mach. Tools Manuf..

[50] You B-H (2009). Passive micro-assembly modular, hot embossed, polymer microfluidic devices using exact constraint design. J. Micromech. Microeng..

[51] You, B. H. *Microassembly Technology For Modular, Polymer Microfluidic Devices* 169. Electronic Thesis and Dissertation Library, The Department of Mechanical Engineering, Louisiana State Univ. (2008).

[52] Whitney DE (1999). Designing assemblies. Res. Eng. Des..

[53] Chen PC (2010). Titer-plate formatted continuous flow thermal reactors: Design and performance of a nanoliter reactor. Sens Actuators B Chem..

[54] Lee TY (2018). Accurate, predictable, repeatable micro-assembly technology for polymer, microfluidic modules. Sens. Actuators B.

[55] Michael DH, Williams PG (1976). The equilibrium and stability of axisymmetric pendent drops. Proc. R. Soc. Ser. A Math., Phys. Sci..

[56] DePaoli DW (1995). Hysteresis in forced oscillations of pendant drops. Phys. Fluids.

[57] Tiggelaar RM (2003). Analysis systems for the detection of ammonia based on micromachined components modular hybrid versus monolithic integrated approach. Sens. Actuators B Chem..

[58] Hasegawa, T. & Ikuta, K. in *Micro Total Analysis Systems 2001* (Monterey, CA, 2001).

[59] Wang H (2012). Fully integrated thermoplastic genosensor for the highly sensitive detection and identification of multi-drug resistant tuberculosis (MDR-TB). Angew. Chem. Int..

[60] Gray, B. L., et al., Mechanical and fluidic characterization of microfluidic interconnects for lab-on-a-chip applications. In *IEEE 14th International Mixed-Signals, Sensors, and System Test Workshop, Vancouver, B.C*. 5 (2008).

[61] Zhao X (2020). Robust, transparent, superhydrophobic coatings using novel hydrophobic/hydrophilic dual-sized silica particles. J. Colloids Interfaces.

