# Supplementary material for: Leakage pressures for gasketless superhydrophobic fluid interconnects for modular lab-on-a-chip systems
Source: Microsyst Nanoeng. 2021 Sep 2;7:69. doi: 10.1038/s41378-021-00287-6 (PMC8433346; doi:10.1038/s41378-021-00287-6)
Supplement: Supplementary file 1 — Supplemental Material [file 41378_2021_287_MOESM1_ESM.pdf]

Nature Microsystems and Nanoengineering

Supplementary Information

for

Leakage Pressures for Gasketless Superhydrophobic Fluid Interconnects (GSFI) for Modular Lab-on-a-Chip Systems

Christopher R. Brown[a,b], Xiaoxiao Zhao[a,b],Taehyun Park[a,b,c], Pin-Chuan Chen[a,b], Byoung Hee You[a,b,d], Daniel S. Park[a,b], Steven A. Soper[e,f], Alison Baird[g], and Michael C. Murphy[a,b]

[a]*Center for Bio-Modular Multi-Scale Systems, Louisiana State University, Baton Rouge, LA, 70803*

[b]*Department of Mechanical & Industrial Engineering, Louisiana State University, Baton Rouge, LA, 70803*

[c]*School of Mechanical Engineering, Kyungnam University, Changwon, South Korea*

[d]*Department of Engineering Technology,Texas State University, San Marcos, TX, 78666*

[e]*Department of Mechanical Engineering, The University of Kansas, Lawrence, KS, 66045*

[f]*Department of Chemistry, The University of Kansas, Lawrence, KS, 66045*

[g] *SUNY Downstate Stroke Center, University Hospital of Brooklyn, Brooklyn, NY, 11203*

Fax:+1 225 578 5924; Tel:+1 225 578 5921; E-mail: murphy@lsu.edu

Table of Contents

For

Supplementary Information

Materials and Methods

Radial Misalignment S3

Leakage Pressure Measurement.. S8

Gap and Misalignment Measurements S10

Results and Discussion

Hydrobead-P® Thickness Measurements S12

Hydrobead-P® Water Contact Angle Measurements S13

Difference between the Leakage Pressure Model and Measurements S14

Assembled Unclamped Chip Image S14

Materials and Methods

<u>Radial Misalignment.</u> The superhydrophobic assembly consisted of two identical polymer chips with through-holes that were aligned and assembled using three pairs of kinematic alignment structures (see Figs. 1(b) and 4(b)). The alignment structures both passively set the gap and concentrically aligned the through holes. Offset measurements from edge alignment standards (see Fig. 1(b) and 4(a)) coupled with a mathematical model quantified the performance of the alignment structures and elucidated the effect of radial misalignment on the maximum pressure capability of the superhydrophobic seal. Cartesian base and feature coordinate frames were defined on each chip (see Fig. S1). Homogeneous coordinate transformation matrices mapped information in the feature coordinates on each chip to the base coordinate system. Similar transformations related features between chips that enabled the coupling of offset measurements along the edge of the assembly to the geometry. A least squares approximation was used to solve the linear system and determine the

Body-fixed Cartesian Coordinate Systems on Test Chips

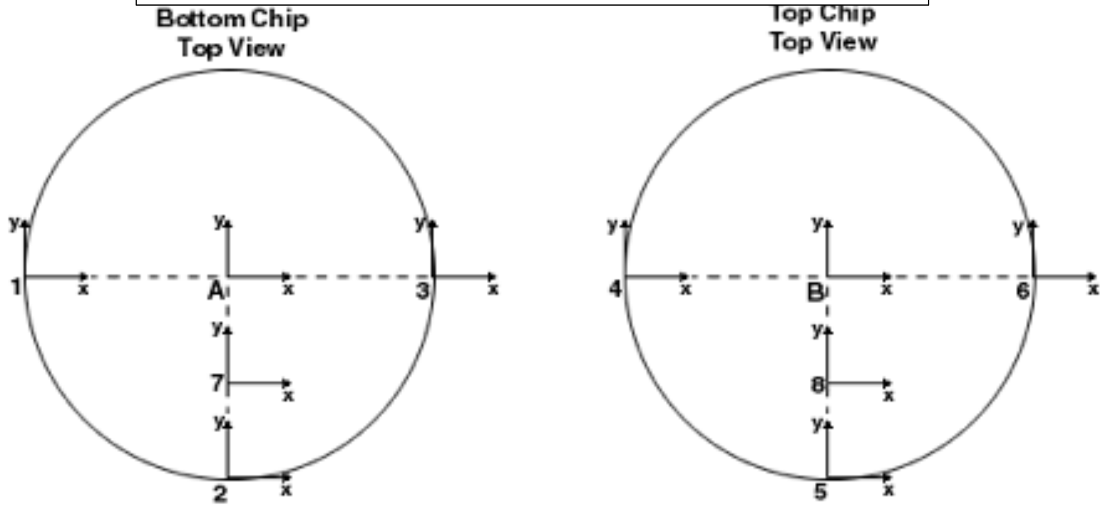

Figure S1. Location of Cartesian base and feature coordinate frames used in the evaluation of the effect of misalignment on the chips forming the superhydrophobic stack.

variation transformation between each chip's base coordinate frame. The variation transformation matrix helped derive the equation for radial misalignment between the superhydrophobic through holes. Each chip was assumed to be a rigid body. The base coordinate system was located at the geometric center of each chip and four feature coordinate frames were located at the left, top, and right alignment standards and the chip's through-hole. Fig. S1 shows the locations of the coordinate frames on the chips. The homogeneous coordinate transformation matrices consisted of a 3 x 3 rotation matrix, R, which described the orientation of the feature coordinate frame with respect to the base coordinates, and a 3 x 1 translation vector, p, which located the origin of the feature coordinates in the base coordinate frame. Eq. S1 shows the general form of the nominal coordinate transformation matrix.

$$T = \begin{bmatrix} R & p \\ 0^T & 1 \end{bmatrix} \qquad \text{[Eq. S1]}$$

The offset measurements collected with an optical microscope at the left, right, and top alignment standards were projections on the x-z and y-z planes. The rigid body assumption coupled the transformations between the assembly's alignment standards to each other (Fig. S1).

The coordinate transformation matrices used to couple the bottom chip's base coordinate frame, A, to the feature coordinate frames, 1, 2, 3 and 7, were identical to the transformations used to connect the top chip's base coordinate frame, B, to its feature coordinate frames, 4, 5, 6 and 8. The top and bottom chip transformations were as shown in Eqs. S2-S5:

$$T_{A1} = \begin{bmatrix} R_{A1} & P_{A1} \\ 0^T & 1 \end{bmatrix} = T_{B4} \qquad \text{[Eq. S2]}$$

$$T_{A3} = \begin{bmatrix} R_{A3} & P_{A3} \\ 0^T & 1 \end{bmatrix} = T_{B6} \qquad \text{[Eq. S3]}$$

$$T_{A2} = \begin{bmatrix} R_{A2} & P_{A2} \\ 0^T & 1 \end{bmatrix} = T_{B5} \qquad \text{[Eq. S4]}$$

$$T_{A7} = \begin{bmatrix} R_{A7} & P_{A7} \\ 0^T & 1 \end{bmatrix} = T_{B8} \quad \text{[Eq. S5]}$$

where the rotation matrix was the identity matrix since the coordinate systems were aligned.

$$R_{A1} = R_{A2} = R_{A3} = R_{A7} = I_3 = \begin{bmatrix} 1 & 0 & 0 \\ 0 & 1 & 0 \\ 0 & 0 & 1 \end{bmatrix} \quad \text{[Eq. S6]}$$

and

$$P_{A1} = [-\overline{A3} \quad 0 \quad 0]^T \quad \text{[Eq. S7]}$$

$$P_{A3} = [\overline{A3} \quad 0 \quad 0]^T \quad \text{[Eq. S8]}$$

$$P_{A2} = [0 \quad -\overline{A2} \quad 0]^T \quad \text{[Eq. S9]}$$

$$P_{A7} = [0 \quad -\overline{A7} \quad 0]^T \quad \text{[Eq. S10]}$$

where $\overline{A3}$ is the distance between the A and 3 coordinate frames (31.459 mm), $\overline{A2}$ is the distance between A and 2 coordinate frames (30.345 mm), and $\overline{A7}$ is the distance between the A and 7 coordinate frames (29.223 mm).

The two rigid body chips were assembled with a nominal gap between them equal to the infinity norm of the assembly's gap measurements. The infinity norm is defined by Eq. S11.

$$\|\boldsymbol{z}_{25}\|_\infty = \max_i |\boldsymbol{z}_{25,\mathrm{i}}| = \varepsilon_z \quad \text{[Eq. S11]}$$

The fixed distance assumption constrains the translation along the z axis and rotation about the x and y axes for the transformation between the A and B coordinate frames. The relationship between the base coordinate frames of the assembly (A and B) can be described by the variation transformation matrix that combines translation along the x and y axes and rotation about the z axis. Eq. S12 describes the variation transformation between the A and B coordinate frames. It assumes small angles of rotation about the z-axis.

$$T_{AB} = trans(\varepsilon_x \quad \varepsilon_y \quad \varepsilon_z) = \begin{bmatrix} 1 & -\delta\theta_z & 0 & \varepsilon_x \\ \delta\theta_z & 1 & 0 & \varepsilon_y \\ 0 & 0 & 1 & \varepsilon_z \\ 0 & 0 & 0 & 1 \end{bmatrix} \quad \text{[Eq. S12]}$$

Homogeneous coordinate transformations were used to map location of the alignment standards on the top and bottom chips into the base coordinates. This framework coupled the measurements to the geometry of the assembly. Eqs. S19-S21 show the variation transformations relating each chips left, right, and top alignment standards.

$$T_{A4} = T_{AB}T_{B4} \quad \text{[Eq. S13]}$$

$$T_{A6} = T_{AB}T_{B6} \quad \text{[Eq. S14]}$$

$$T_{A5} = T_{AB}T_{B5} \quad \text{[Eq. S15]}$$

$$T_{A1}T_{14} = T_{A4} \quad \text{[Eq. S16]}$$

$$T_{A3}T_{36} = T_{A6} \quad \text{[Eq. S17]}$$

$$T_{A2}T_{25} = T_{A5} \quad \text{[Eq. S18]}$$

$$T_{14} = {T_{A1}}^{-1}T_{A4} = \begin{bmatrix} 1 & -\delta\theta_z & 0 & \varepsilon_x \\ \delta\theta_z & 1 & 0 & \varepsilon_y - \overline{A3}(\delta\theta_z) \\ 0 & 0 & 1 & \varepsilon_z \\ 0 & 0 & 0 & 1 \end{bmatrix} \quad \text{[Eq. S19]}$$

$$T_{36} = {T_{A3}}^{-1}T_{A6} = \begin{bmatrix} 1 & -\delta\theta_z & 0 & \varepsilon_x \\ \delta\theta_z & 1 & 0 & \varepsilon_y + \overline{A3}(\delta\theta_z) \\ 0 & 0 & 1 & \varepsilon_z \\ 0 & 0 & 0 & 1 \end{bmatrix} \quad \text{[Eq. S20]}$$

$$T_{25} = {T_{A2}}^{-1}T_{A5} = \begin{bmatrix} 1 & -\delta\theta_z & 0 & \varepsilon_x + \overline{A2}(\delta\theta_z) \\ \delta\theta_z & 1 & 0 & \varepsilon_y \\ 0 & 0 & 1 & \varepsilon_z \\ 0 & 0 & 0 & 1 \end{bmatrix} \quad \text{[Eq. S21]}$$

The average of the measurements at the left alignment standards, y_{14} estimated the y-offset of the

edge projected on to the bottom chip's y-z plane. Similarly, the averages of the right and top alignment standards estimate the y- and x-offset of the edge projected on the bottom chip's y-z and x-z planes. Eqs. S22-S24 show the offset measurements sample means.

$$\overline{\boldsymbol{y_{14}}} = \frac{\sum_0^n y_{14,i}}{n}$$ [Eq. S22]

$$\overline{\boldsymbol{y_{36}}} = \frac{\sum_0^n y_{36,i}}{n}$$ [Eq. S23]

$$\overline{\boldsymbol{x_{25}}} = \frac{\sum_0^n y_{25,i}}{n}$$ [Eq. S24]

Eqs. S22-S24 can be equated to the appropriate components of the coordinate transformations in Eqs. S19-S21 to derive Eqs. S25-S27

$$\overline{\boldsymbol{y_{14}}} = \varepsilon_y - \overline{A3}(\delta\theta_z)$$ [Eq. S25]

$$\overline{\boldsymbol{y_{36}}} = \varepsilon_y + \overline{A3}(\delta\theta_z)$$ [Eq. S26]

$$\overline{\boldsymbol{x_{25}}} = \varepsilon_x + \overline{A2}(\delta\theta_z)$$ [Eq. S27]

Eqs. S25-S27 can be written as a linear system $A\boldsymbol{x} = \boldsymbol{b}$ as follows (Eq. S28):

$$\begin{bmatrix} 1 & 0 & \overline{A2} \\ 0 & 1 & -\overline{A3} \\ 0 & 1 & \overline{A3} \end{bmatrix} \begin{bmatrix} \varepsilon_x \\ \varepsilon_y \\ \delta\theta_z \end{bmatrix} = \begin{bmatrix} \overline{\boldsymbol{x_{25}}} \\ \overline{\boldsymbol{y_{14}}} \\ \overline{\boldsymbol{y_{36}}} \end{bmatrix}$$ [Eq. S28]

where a unique solution for **x** exists if **b** is in the colspace(A) and the dim[colspace(A)]=n. Since **b** is an estimate of the actual offsets at the edge and contain error, there is a probability that **b** will not be in the colspace(A) and therefore the equations would not have a solution. However, the system of equations will always have an approximate least squares solution. Eqs. S29-S32 show the derivation using of the least squares solution.

$$A\boldsymbol{x} = \boldsymbol{b}$$ [Eq. S29]

$$A^T A\boldsymbol{x}^* = A^T\boldsymbol{b}$$ [Eq. S30]

$$A^+ = (A^T A)^{-1} A^T$$ [Eq. S31]

$$\boldsymbol{x}^{*} = A^{+}\boldsymbol{b} \quad \text{[Eq. S32]}$$

where $\boldsymbol{x}^{*}$ is the least squares approximation of $[\varepsilon_x\ \varepsilon_y\ \delta\theta_z]$, A^{+} is the pseudoinverse of A, and **b** is the averages of the measurements at the alignment standards. Eq. S32 and Eq. S12 give the coordinate transformation that connects the base coordinate frame of the bottom chip, A, with the base coordinate frame of the top chip, B (Fig. S8). Using similar coordinate transformations, this least squares approximation can be translated to the coordinate frames connecting the top and bottom chips through holes.

$$T_{A7}T_{78} = T_{A8} \quad \text{[Eq. S33]}$$

$$T_{A8} = T_{AB}T_{B8} \quad \text{[Eq. S34]}$$

$$T_{78} = T_{A7}{}^{-1}T_{A8} \quad \text{[Eq. S35]}$$

The x and y translation components from Eq. S35 give the radial misalignment between the through holes.

Leakage Pressure Measurement The leakage pressure experimental apparatus consisted of two branches, the pressurized liquid column, and the microfluidic system. The pressurized liquid column was upstream of the microfluidic system. A simplified schematic of the pressure measurement system is shown in Fig. S2. Ultra high purity nitrogen (1) was regulated to 172 kPa (25 psi) by its cylinder regulator (2) then passed through a 20 μm line filter (3) before it was regulated to a pressure from 0-34.47 kPa (0-5 psi) by a dual valve pressure controller (68027-60, Cole-Parmer, Vernon Hills, IL) (4). The regulated nitrogen flowed through a 9.525 mm (3/8 in) DIA NPT bronze tee junction with a 172 kPa (25 psi) ASME-Code Brass Pop-Safety Valve (5) to the top of a pressurized liquid column (6). Pressurized deionized water exited the bottom of the column and was transported through a 9.525 mm (3/8 in) DIA NPT bronze 3-way valve to a ball valve (P-732 Microfluidic Ball Valve, Upchurch Scientific, Oak Harbor, WA) (7). A tee

Schematic of Leakage Pressure Measurement Apparatus

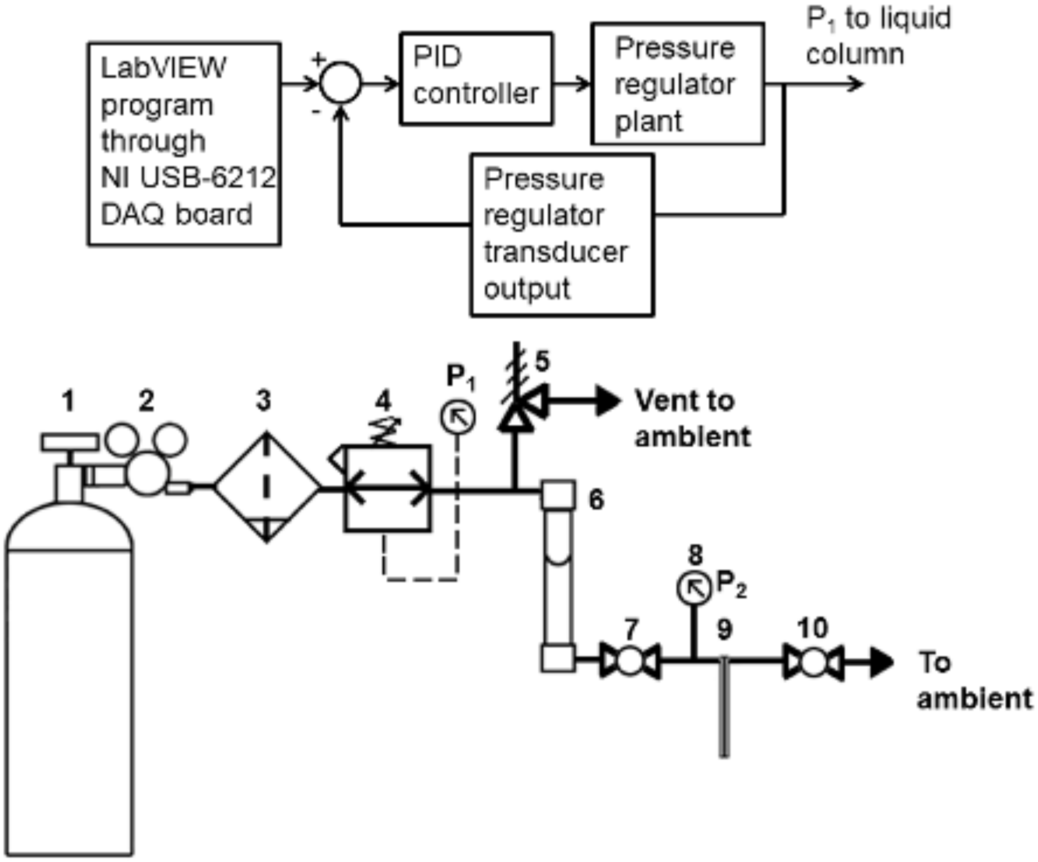

Fig. S2. A schematic of the experimental apparatus and a block diagram of the control system. The experimental apparatus components include 1) ultra high purity nitrogen cylinder, 2) nitrogen cylinder regulator, 3) 5 μm particle filter, 4) Cole-Parmer 0-5 psi dual valve pressure controller (Vernon Hills, IL), 5) 0-25 psi pressure relief valve, 6) PVC pressurized liquid column, 7) Upchurch Scientific P-732 upstream microfluidic ball valve (Oak Harbor, WA), 8) Honeywell ASDX pressure transducer (Golden Valley, MN), 9) interconnect assembly, 10) P-732 downstream microfluidic ball valve. Additionally, a National Instruments (Austin, TX) USB-6212 data acquisition board in conjunction with a LabVIEW program sent a set point signal to the pressure controller and received pressure readings from the pressure controller and the pressure transducer.

junction (P-727, Upchurch Scientific) was connected to either a 0-6.89 kPa (0-1 psi) (ASDXAVX001PGAA5) or a 0-34.47 kPa (0-5 psi) (ASDXAVX005PGAA5) pressure transducer (Honeywell S&C, Golden Valley, MN) (8) and the interconnect assembly (9). The fluid exited the interconnect assembly and flowed through a downstream ball valve (P-732 Microfluidic Ball Valve, Upchurch Scientific) (10) and out of the system into a dish.

A custom LabVIEW 2012 (National Instruments, Austin, TX) program running on a laptop computer was used to provide a graphical user interface (GUI) for the system. A data acquisition board (NI USB-6212 DAQ, National Instruments, Austin, TX) with 16 dual-sided A/D inputs, 2 analog outputs, and 32 digital I/O channels with a maximum sampling rate of 4 kHz was connected to the computer through a USB port. The GUI displayed the system inputs, pressure measurements, and the system output, the pressure controller set point. There were two input signals, from the rupture pressure (P_2 in Fig. S2) sensor and the fluid column pressure controller transducer (P_1 in Fig. S2), and one output signal, going to the fluid column pressure controller.

Pressure transducers (P_2 in Fig. S2) with two different ranges were used depending on the pressure capability of the gasketless seal: (1) a 0-6.89 kPa (0-1 psi) sensor for lower pressures with an accuracy of ±0.138 kPa; and (2) a 0-34.47 kPa (0-5 psi) sensor for the higher pressures with an accuracy of ±0.689 kPa. Both pressure transducers had a time constant of 1 ms. The fluid column pressure controller (4 in Fig. S2) had a pressure transducer accuracy of ±0.034 kPa and the controller had a set point accuracy of ±0.086 kPa.

The leakage pressure measurement had a start-up procedure, a priming procedure, and a rupture pressure testing procedure. To prime the system, upstream (7 in Fig. S2) and downstream ball valves (10 in Fig. S2) were opened, connecting the gasketless seal assembly through the microfluidic tee to the deionized water column (6 in Fig. S2), pressurized to 0.345 kPa (0.05 psi), until the liquid bridge was formed, establishing the seal. With the system ready, the upstream and downstream ball valves were closed and the liquid column pressure was set to 0 kPa (0 psi). For the leakage pressure tests, the upstream ball valve was opened and the pressure increased across the seal incrementally until rupture was detected. Once leakage was observed, the upstream microfluidic ball valve (7 in Fig. S2) was closed, and the LabVIEW program halted.

Gap and Misalignment Measurements. The gap and misalignment were measured using the Nikon Measurescope MM-11 (Melville, NY) with a Diagnostic Instruments, Inc microscope

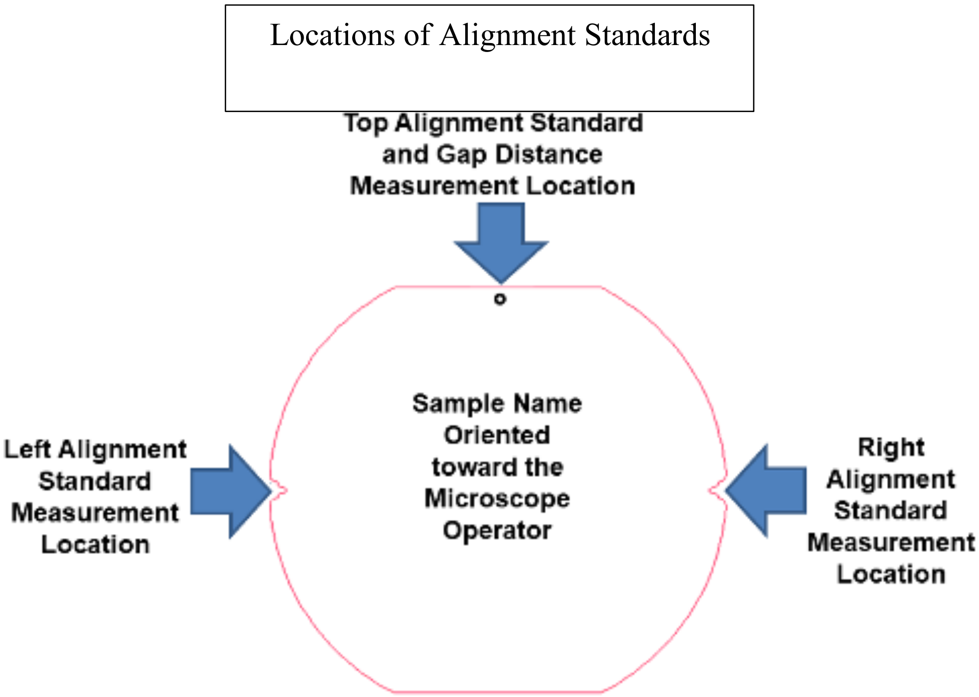

Fig. S3. Overview of the gap distance and misalignment measurement locations on the interconnect assembly.

camera (Sterling Heights, MI) with SPOT advanced imaging software, and a QUADRA-CHEK 2000 (Metronics, Schaumburg, IL). The gap, the left alignment standard, and the right alignment standard were each measured ten times for every interconnect assembly. The top alignment standard was measured ten times for forty-six of the sample assemblies. The radial misalignment was calculated using the method outlined in the first section (pp. S3-S8). The location of the gap measurements and the left, right, and top misalignment measurements are shown in Fig. S3. Fig. S4

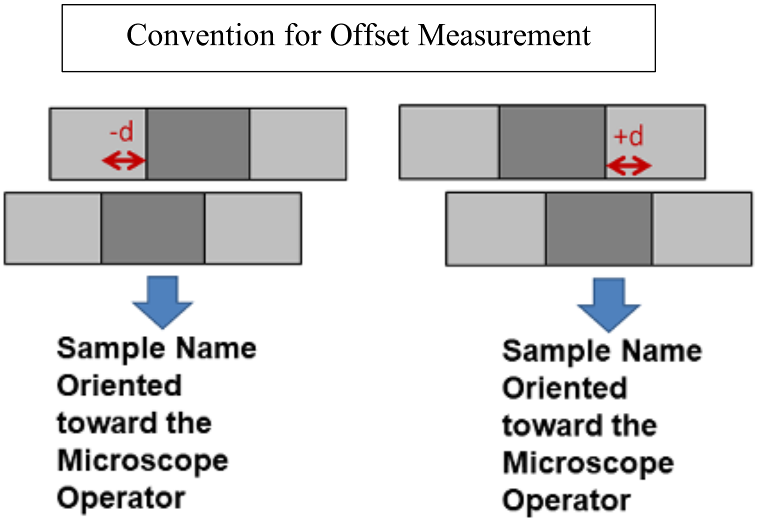

Fig. S4. The convention adopted for the offset direction for the right, left, and top alignment measurements.

displays the convention adopted for the misalignment measurements. Both the gap and misalignment measurements had a start-up procedure and a data collection procedure.

Results and Discussion

Hydrobead-P® Thickness Measurements. Thickness measurements of Hydrobead-P® (Hydrobead-P® "old formula", Hydrobead, San Diego, CA) were conducted using a Nikon Measurescope MM-11 (Melville, NY) with a height gage. A reference elevation was established by using Scotch™ tape (3M, St. Paul, MN) to mask a portion of the sample chip from the center of the chip to the edge during spin coating and curing. Twelve sample chips were selected at random to be masked and then spin coated with Hydrobead-P® and then cured for one hour in the VWR 1602 (Radnor, PA) oven preheated to 100°C. After curing, the tape was removed with

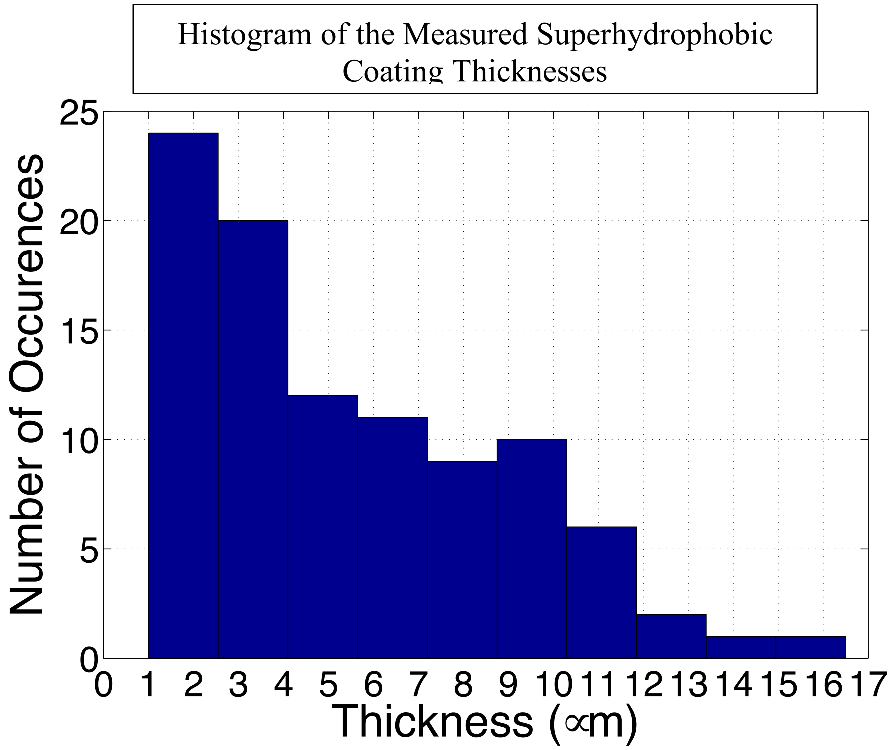

Fig. S5. A histogram of the measured Hydrobead-P® thicknesses across twelve spin coated samples using the optical microscope.

tweezers and the thickness of the Hydrobead-P® was measured using the optical microscope by focusing on the reference surface, zeroing the height gage, and then focusing on the peaks of the adjacent Hydrobead-P®. The resulting number on the height gage was recorded as the thickness of the coating. For each sample, the two long tape edges were each measured four times and the tape edge closest to the chip's flat was measured two times. The average measured thickness was 5.58 μm with a standard deviation of 3.41 μm. Fig. S5 shows a histogram of the resulting ninety-six thickness measurements across twelve spin coated samples.

<u>Hydrobead-P® Water Contact Angle Measurements.</u> The water contact angle indicated the wettability of the Hydrobead-P® surface and was used as a parameter in the maximum rupture

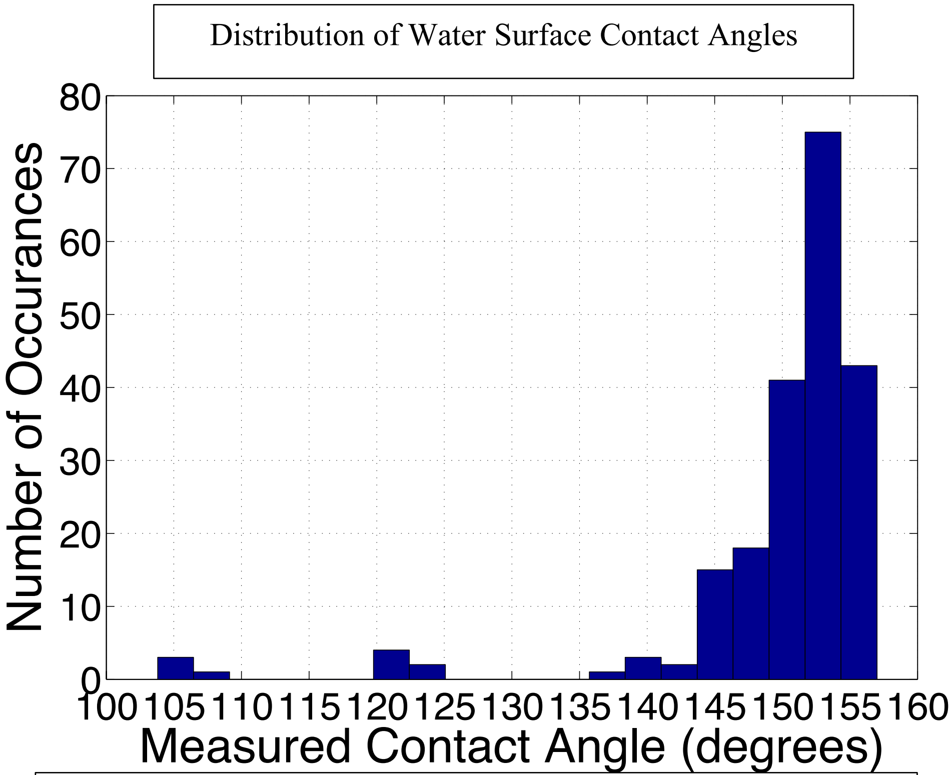

Fig. S6. A histogram of the 208 contact angle measurements of the spin coated Hydrobead-P® surface using the sessile drop method with a VCA Optima (Billerica, MA).

Fig. S6. A histogram of the 208 contact angle measurements of the spin coated Hydrobead-P® surface using the sessile drop method with a VCA Optima (Billerica, MA).

pressure model. To measure the contact angle, thirteen injection molded COC samples were spin coated with Hydrobead-P® (Hydrobead-P® "old formula", Hydrobead, San Diego, CA) and cured for 1 hour in the VWR 1602 (Radnor, PA) oven preheated to 100°C. The contact angle of each sample was measured sixteen times across the surface using the sessile drop technique with a VCA Optima (Billerica, MA). The average contact angle was 149.8° and the standard deviation was 8.74°. Fig. S6 is a histogram of the 208 contact angle measurements.

Assembled Chips An example of an unclamped assembly is shown in Fig. S7.

Fully Assembled Chips with World-to-Chip Connectors

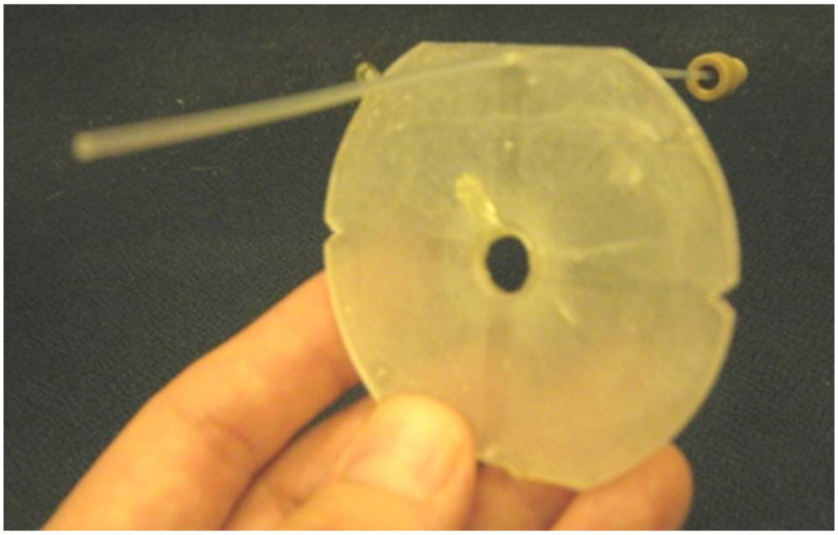

Fig. S7. A photograph of a pair of assembled chips with world-to-chip connection and superhydrophobic surfaces coated.
